# Development and validation of parental knowledge, attitude and practice in eye problem among children questionnaire (PEPC-KAPQ)

**DOI:** 10.1371/journal.pone.0291062

**Published:** 2023-09-08

**Authors:** Nor Diyana Hani Ghani, Mohd Harimi Abd Rahman, Norliza Mohamad Fadzil, Zainora Mohammed, Hanif Farhan Mohd Rasdi, Nur Syafiqah Shafie

**Affiliations:** 1 Optometry and Vision Science Program, Centre for Rehabilitation and Special Needs Study (iCaRehab), Faculty of Health Sciences, Universiti Kebangsaan Malaysia, Kuala Lumpur, Malaysia; 2 Occupational Therapy Program, Centre for Rehabilitation and Special Needs Study (iCaRehab), Faculty of Health Sciences, Universiti Kebangsaan Malaysia, Kuala Lumpur, Malaysia; University of Hafr Al-Batin, SAUDI ARABIA

## Abstract

**Background:**

Most eye problems among children can be detected and treated at an early age to reduce the prevalence of visual impairment. Understanding the knowledge, attitude, and practice (KAP) among parents about common children’s eye problems is fundamental to encourage parents to seek early eye care services for their children. This study aims to develop a Parental Knowledge, Attitude, and Practice in Eye Problem among Children Questionnaire (PEPC-KAPQ) and evaluate its psychometric properties.

**Methods:**

This study involved developing a questionnaire and was conducted in Kuala Lumpur, Malaysia from July 2021 until June 2022. The questionnaire was developed based on a literature review and expert consultation. The first phase includes a systematic literature review to generate the items for the questionnaire. A group of five panels was then invited to perform content validity for the questionnaire. Face validity was conducted among ten parents to get feedback for the questionnaire. Construct validity and reliability of the questionnaire were measured by which the questionnaire was administered to a total of 134 parents and 64 parents for reliability test.

**Result:**

The final PEPC-KAPQ consists of four main sections: demographic, knowledge, attitude, and practice with 52 items. The content validity index was 0.85 for all domains of KAP. Modified kappa showed excellent value for most items for all domains. The Kaiser-Meyer-Olkin sampling adequacy showed acceptable scores of 0.84, and Bartlett’s Test of Sphericity was significant (x^2^ = 3172.09, p<0.0001). Kuder-Richardson-2 of the domain knowledge was 0.95. Cronbach’s *α* coefficient of domain attitude and practice were 0.92 and 0.88, respectively and the intraclass correlation of domain attitude and practice were 0.93 and 0.94 respectively. Bland and Altman’s plots show that majority of the data fell within the limits of agreement.

**Conclusion:**

The findings of this validation and reliability study show that the developed questionnaire has a satisfactory psychometric property for measuring the KAP of parents regarding eye problems among children.

## Introduction

Globally, visual impairment is a significant health problem that affects people of all ages [1]. According to the World Health Organization (WHO), 29% of the 36 million people with vision problems are children [2]. There are 80% of the cause of vision problems in children that can be treated if detected early [3]. Early detection and treatment are crucial as treatment delay negatively influences children’s development, learning, communication, health, occupational performance and quality of life [4]. This is only achievable if the parents are concerned about their child’s eye health care because they are the primary caregiver and are responsible for ensuring that their children receive early care [5].

In order to create awareness among parents, insight into the gaps in parents’ knowledge, attitude, and practice (KAP) regarding common eye problems in children is important. Although there were a few studies to determine the level of KAP, the study focused on specific eye diseases only and none focused on children’s common vision problems [6–11]. Furthermore, some studies only assess two components of the KAP [12–15]. In general, there is a lack of validated KAP instruments focusing on parents regarding eye problems among children.

This study aims to develop a Parental KAP in Eye Problems among Children Questionnaire (PEPC-KAPQ) and evaluate its psychometric properties. The information obtained can be used to develop and implement a better awareness and educational program for the specific population to improve the level of KAP among parents.

## Materials and methods

The study was approved by the Research Ethics Committee, Universiti Kebangsaan Malaysia (JEP-2020-739). This study involved developing a questionnaire and was conducted in Kuala Lumpur, Malaysia from July 2021 until June 2022. The questionnaire was developed and validated via the following steps [16–19], including item generation, content validity by panels, face validity, construct validity and reliability of the final version of the KAP questionnaire and test-retest reliability. For this study, purposive sampling was used for content validity, convenience sampling was used for face validity, and random sampling was used for construct validity and reliability. Signed informed consent was obtained from all subjects.

### Item generation

A systematic literature review was conducted to generate the question included in the KAP questionnaire. "MeSH" terms such as eye health, knowledge, attitude, practice, and children were used. Previous studies on current eye problems among children were searched through the EBSCOhost, MEDLINE, and Scopus [19]. Related papers were selected, and relevant questions were generated and constructed [16–19].

Following the literature review, the primary investigator conducted two sessions of focus group discussion involving five participants, which consisted of 4optometrists and 1 orthoptic to obtain the information and ideas to construct the KAP questionnaire. Spontaneous information was obtained from the participants. If the expert panels agreed with how the items were constructed, more specific questions were asked. The data were qualitatively analyzed, and new items were added to the questionnaire.

A draft set of questionnaires were created, ensuring that no items were duplicated. The items were written in a simple Malay language that the participants could understand, refer to a single idea expressed in the first person and avoid double negatives. For domain knowledge, a dichotomous scale as either yes or no was used as a unidimensional response option to collect direct and precise responses [17]. In contrast, a 4-point Likert scale was used for domain attitude and practice to avoid a neutral point [20, 21].

### Content validity

After constructing the draft questionnaire, a team of five panels validates the questionnaire for critical assessment, input, and content validity in terms of relevancy and clarity. This is to ensure the legitimacy of the content of the draft questionnaire. The panels included one ophthalmologist (minimum of five years’ experience in the field of ophthalmology), one optometrist (minimum of five years’ experience in the field of optometry), one teacher (minimum of five years’ experience in the field of education) and two parents (minimum with one child ages 7 to 12 years old). For the content validity index (CVI), the panels were asked to rate each scale item on the construct question’s relevancy and clarity [22, 23]. A 4-point scale was used to avoid a neutral point [20, 21]. The panels were also requested to express their thought or ideas on the items by filling in the comment section in the questionnaire.

The necessary modification was made based on the panel’s opinion and suggestions. Items with an I-CVI of ≥0.78 remained, I-CVI of 0.70–0.78 were revised, and those ≤0.70 were eliminated [23]. The scale-level content validity index based on average methods (S-CVI/Ave), scale-level content validity index based on universal agreement method (S-CVI/UA), probability of change agreement (P_c_), and Modified kappa (K) were calculated [22, 23]. The preliminary version of the questionnaire (pre-PEPC-KAPQ) was developed and proceeded for face validity.

### Face validity

The pre-PEPC-KAPQ was pretested on ten parents who met the inclusion criteria and gave consent voluntarily. Inclusion criteria were fluent in Malay and having a child ages 7 to 12 years old. This face validity was conducted to obtain feedback from a convenience sample of parents [16–19] and to determine if the participants’ responses to the question were clear and had no misinterpretation. The parents completed the questionnaire and provided feedback. A minor change was made in the questionnaire if there are any comments.

### Construct validation

The sample size was calculated based on Cochran’s sample size calculation [24];

$$n_{0}=Z^{2}.pq/e^{2}$$

where *n*_0_ is the sample size, *Z*^2^ is the abscissa of the normal curve that cuts off an area *α* at the tails (1.96 for 95% confidence level), *e* is the desired level of precision (0.1), *p* is the estimated proportion of 66.4% from the 14,156 population of household based on *Kajian Penduduk Keluarga Malaysia Kelima* (The Fifth Malaysian Population Survey) [25], and *q* is 1- *p*. The calculated sample size was added with a 20% dropout. Hence, the sample size required was 103. In this study, 200 parents were recruited and 134 parents who met the study design and inclusion criteria were invited to complete the preliminary questionnaire to determine the construct validity and internal consistency. Inclusion criteria were fluent in Malay and having a child ages 7 to 12 years old.

Data were collected from March 2021 to July 2021. Exploratory factor analysis (EFA) was used to test the hypothesized domain structure and examine its substructure, and internal consistency was evaluated using Cronbach’s *α* coefficient. The Kaiser-Meyer-Olkin (KMO) and Bartlett’s sphericity test were used for the EFA to determine the sampling adequacy. These two tests allow an assessment of the suitability of conducting factor analysis. If the KMO value is more significant than 0.50 and Bartlett’s sphericity test value is p<0.05, the data are suitable for factor analysis [16, 18, 26]. A principal component factor analysis using varimax rotation by fixing the number of factors to extract [27]. A cut-off points of 0.3 was used for commonalities, with ideal communalities being 0.7 or above [27].

### Reliability

In this study, the internal consistency (IC) of the items was measured by using Kuder-Richardson-20 (KR-20) and Cronbach’s *α* coefficient [18]. KR-20 was used to evaluate the scale for domain knowledge and Cronbach’s *α* coefficient for the four-point Likert scale for domain attitude and practice. KR-20 coefficient of ≥0.50 and Cronbach’s *α* coefficient ≥0.70 was considered satisfactory internal consistency evidence for the constructed questionnaire [18, 28].

Test-retest reliability was performed to analyze the stability and consistency of the questionnaire over time by using the level of agreement, Bland and Altman plot, and standard error of measurement. The sample size calculation was done by using a web-based sample size calculator for reliability study, where for the test-retest reliability of a measurement tool by Intraclass correlation (ICC) is expected 0.85, the lowest acceptable ICC is 0.70, the significant level *α* is 0.50 and a power of 80%. The dropout rate expected is 10% [29]. Hence, the sample size required was 59 and 64 parents who met the inclusion criteria from the construction validity were invited to complete the KAP questionnaire within a one-month interval, which is considered sufficient time to avoid recall bias due to the memory effect [30].

## Results

### Item generation

A systematic literature search shows 622 articles using the related keywords, and 46 are relevant to our study. The relevant article was analyzed, and 140 items were generated for the questionnaire based on three domains: knowledge, attitude, and practice. The FGD with the panel led to the elimination of 34 items as those items were hard to understand and 45 items were removed during the subsequent FGD because of overlapping questions. There are 61 items in the questionnaire draft, with 28 items for domain knowledge, 20 for domain attitude and 13 for domain practice.

### Content validity

Five panels evaluated the content validity of the draft questionnaire for domain knowledge, attitude, and practice. The content validity index (I-CVI), scale-level content validity index based on average methods (S-CVI/Ave), scale-level content validity index based on universal agreement method (S-CVI/UA), probability of change agreement (P_c_), and Modified kappa (K) was calculated for each domain based on relevancy and clarity (Tables 1 and 2). Based on the result and comments of the panel, two items for domain knowledge with the I-CVI value of 0.40 and 0.60 and k<0.50 were eliminated. The final draft of the questionnaire consists of 26 items for domain knowledge, 20 items for domain attitude and 13 items for domain practice.

**Table 1:** The relevance rating of each item by five panels.

Item	Panel in agreement	I-CVI	UA	P_c_	Kappa	Interpretation	S-CVI/Ave	S-CVI/UA
K_1	5	1.00	1	0.03	1.00	Excellent	0.91	0.85
K_2	4	0.80	0	0.16	0.76	Good		
K_3	5	1.00	1	0.03	1.00	Excellent		
K_4	5	1.00	1	0.03	1.00	Excellent		
K_5	5	1.00	1	0.03	1.00	Excellent		
K_6	5	1.00	1	0.03	1.00	Excellent		
K_7	5	1.00	1	0.03	1.00	Excellent		
K_8	4	0.80	0	0.16	0.76	Good		
K_9	5	1.00	1	0.03	1.00	Excellent		
K_10	4	0.80	0	0.16	0.76	Good		
K_11	4	0.80	0	0.16	0.76	Good		
K_12	3	0.60	0	0.31	0.42	Fair*		
K_13	2	0.40	0	0.31	0.13	Poor*		
K_14	5	1.00	1	0.03	1.00	Excellent		
K_15	4	0.80	0	0.16	0.76	Good		
K_16	5	1.00	1	0.03	1.00	Excellent		
K_17	5	1.00	1	0.03	1.00	Excellent		
K_18	5	1.00	1	0.03	1.00	Excellent		
K_19	5	1.00	1	0.03	1.00	Excellent		
K_20	4	0.80	0	0.16	0.76	Good		
K_21	5	1.00	1	0.03	1.00	Excellent t		
K_22	4	0.80	0	0.16	0.76	Good		
K_23	5	1.00	1	0.03	1.00	Excellent		
K_24	4	0.80	0	0.16	0.76	Good		
K_25	5	1.00	1	0.03	1.00	Excellent		
K_26	5	1.00	1	0.03	1.00	Excellent		
K_27	5	1.00	1	0.03	1.00	Excellent		
K_28	5	1.00	1	0.03	1.00	Excellent		
A_1	4	0.80	0	0.16	0.76	Excellent	0.95	0.85
A_2	4	0.80	0	0.16	0.76	Excellent		
A_3	5	1.00	1	0.03	1.00	Excellent		
A_4	4	0.80	0	0.16	0.76	Excellent		
A_5	5	1.00	1	0.03	1.00	Excellent		
A_6	5	1.00	1	0.03	1.00	Excellent		
A_7	4	0.80	0	0.16	0.76	Excellent		
A_8	5	1.00	1	0.03	1.00	Excellent		
A_9	5	1.00	1	0.03	1.00	Excellent		
A_10	4	0.80	0	0.16	0.76	Excellent		
A_11	5	1.00	1	0.03	1.00	Excellent		
A_12	5	1.00	1	0.03	1.00	Excellent		
A_13	5	1.00	1	0.03	1.00	Excellent		
A_14	5	1.00	1	0.03	1.00	Excellent		
A_15	5	1.00	1	0.03	1.00	Excellent		
A_16	5	1.00	1	0.03	1.00	Excellent		
A_17	5	1.00	1	0.03	1.00	Excellent		
A_18	5	1.00	1	0.03	1.00	Excellent		
A_19	5	1.00	1	0.03	1.00	Excellent		
A_20	5	1.00	1	0.03	1.00	Excellent		
P_1	5	1.00	1	0.03	1.00	Excellent	0.97	0.85
P_2	5	1.00	1	0.03	1.00	Excellent		
P_3	5	1.00	1	0.03	1.00	Excellent		
P_4	5	1.00	1	0.03	1.00	Excellent		
P_5	4	0.80	0	0.16	0.76	Excellent		
P_6	5	1.00	1	0.03	1.00	Excellent		
P_7	5	1.00	1	0.03	1.00	Excellent		
P_8	5	1.00	1	0.03	1.00	Excellent		
P_9	5	1.00	1	0.03	1.00	Excellent		
P_10	5	1.00	1	0.03	1.00	Excellent		
P_11	5	1.00	1	0.03	1.00	Excellent		
P_12	4	0.80	0	0.16	0.76	Excellent		
P_13	5	1.00	1	0.03	1.00	Excellent		

S-CVI/Ave = 0.978 (accepted), S-CVI/UA = 0.85 (accepted).

*Data was excluded.

**Table 2:** The clarity rating of each item by five panels.

Item	Panel in agreement	I-CVI	UA	P_c_	Kappa	Interpretation	S-CVI/Ave	S-CVI/UA
K_1	5	1.00	1	0.03	1.00	Excellent	0.91	0.85
K_2	4	0.80	0	0.16	0.76	Good		
K_3	5	1.00	1	0.03	1.00	Excellent		
K_4	5	1.00	1	0.03	1.00	Excellent		
K_5	5	1.00	1	0.03	1.00	Excellent		
K_6	5	1.00	1	0.03	1.00	Excellent		
K_7	5	1.00	1	0.03	1.00	Excellent		
K_8	4	0.80	0	0.16	0.76	Good		
K_9	5	1.00	1	0.03	1.00	Excellent		
K_10	4	0.80	0	0.16	0.76	Good		
K_11	4	0.80	0	0.16	0.76	Good		
K_12	3	0.60	0	0.31	0.42	Fair*		
K_13	2	0.40	0	0.31	0.13	Poor*		
K_14	5	1.00	1	0.03	1.00	Excellent		
K_15	4	0.80	0	0.16	0.76	Good		
K_16	5	1.00	1	0.03	1.00	Excellent		
K_17	5	1.00	1	0.03	1.00	Excellent		
K_18	5	1.00	1	0.03	1.00	Excellent		
K_19	5	1.00	1	0.03	1.00	Excellent		
K_20	4	0.80	0	0.16	0.76	Good		
K_21	5	1.00	1	0.03	1.00	Excellent		
K_22	4	0.80	0	0.16	0.76	Good		
K_23	5	1.00	1	0.03	1.00	Excellent		
K_24	4	0.80	0	0.16	0.76	Good		
K_25	5	1.00	1	0.03	1.00	Excellent		
K_26	5	1.00	1	0.03	1.00	Excellent		
K_27	5	1.00	1	0.03	1.00	Excellent		
K_28	5	1.00	1	0.03	1.00	Excellent		
A_1	4	0.80	0	0.16	0.76	Excellent	0.95	0.85
A_2	4	0.80	0	0.16	0.76	Excellent		
A_3	5	1.00	1	0.03	1.00	Excellent		
A_4	4	0.80	0	0.16	0.76	Excellent		
A_5	5	1.00	1	0.03	1.00	Excellent		
A_6	5	1.00	1	0.03	1.00	Excellent		
A_7	4	0.80	0	0.16	0.76	Excellent		
A_8	5	1.00	1	0.03	1.00	Excellent		
A_9	5	1.00	1	0.03	1.00	Excellent		
A_10	4	0.80	0	0.16	0.76	Excellent		
A_11	5	1.00	1	0.03	1.00	Excellent		
A_12	5	1.00	1	0.03	1.00	Excellent		
A_13	5	1.00	1	0.03	1.00	Excellent		
A_14	5	1.00	1	0.03	1.00	Excellent		
A_15	5	1.00	1	0.03	1.00	Excellent		
A_16	5	1.00	1	0.03	1.00	Excellent		
A_17	5	1.00	1	0.03	1.00	Excellent		
A_18	5	1.00	1	0.03	1.00	Excellent		
A_19	5	1.00	1	0.03	1.00	Excellent		
A_20	5	1.00	1	0.03	1.00	Excellent		
P_1	5	1.00	1	0.03	1.00	Excellent	0.97	0.85
P_2	5	1.00	1	0.03	1.00	Excellent		
P_3	5	1.00	1	0.03	1.00	Excellent		
P_4	5	1.00	1	0.03	1.00	Excellent		
P_5	4	0.80	0	0.16	0.76	Excellent		
P_6	5	1.00	1	0.03	1.00	Excellent		
P_7	5	1.00	1	0.03	1.00	Excellent		
P_8	5	1.00	1	0.03	1.00	Excellent		
P_9	5	1.00	1	0.03	1.00	Excellent		
P_10	5	1.00	1	0.03	1.00	Excellent		
P_11	5	1.00	1	0.03	1.00	Excellent		
P_12	4	0.80	0	0.16	0.76	Excellent		
P_13	5	1.00	1	0.03	1.00	Excellent		

S-CVI/Ave = 0.978 (accepted), S-CVI/UA = 0.85 (accepted).

*Data was excluded.

### Face validity

Ten parents complete the preliminary draft questionnaire in 10–15 minutes with no items non-response. The result shows that the questionnaire was considered simple and easy to understand. All 59 items remained without any minor changes and elimination since there was no comment or suggestion from the participants.

### Construct validity

A cross-sectional survey of 134 parents was conducted in construct validity. The mean age of the subjects was 38.25±5.85 years old. There were 113 females (84.3%) and 21 males (15.7%), with 131 (97.8%) of the subject being Malay.

The EFA explored the appropriate construct for domain attitude and practice. Based on the EFA result, the KMO shows 0.84, and Bartlett’s sphericity test was less than 0.001, indicating that the data were suitable for factor analysis. The communalities of each attribute confirmed the validity. All the items with factor loading >0.30 were accepted. For domain attitude, items 9, 11, and 12 were excluded, while items 3, 4, 9, and 10 were excluded from domain practice, as shown in Table 3. The final questionnaire consists of 52 items, with 26 items for domain knowledge, 17 for domain attitude and 9 for domain practice.

**Table 3:** The result of exploratory factor analysis.

Factor	Item	Factor Loading ^a^	Cronbach’s *α*
Attitude	1	0.45	0.92
	2	0.52	
	3	0.48	
	4	0.61	
	5	0.55	
	6	0.61	
	7	0.51	
	8	0.32	
	9	0.26*	
	10	0.31	
	11	0.24*	
	12	0.29*	
	13	0.47	
	14	0.37	
	15	0.37	
	16	0.35	
	17	0.58	
	18	0.57	
	19	0.61	
	20	0.60	
Practice	1	0.64	0.88
	2	0.50	
	3	0.15*	
	4	0.26*	
	5	0.59	
	6	0.35	
	7	0.56	
	8	0.44	
	9	0.08*	
	10	0.07*	
	11	0.59	
	12	0.57	
	13	0.36	

^a^ Extraction method: principal component analysis.

*Data was excluded.

### Reliability

The domain knowledge, KR-20 shows 0.95, and Cronbach’s *α* for domain attitude and practice was 0.92 and 0.88, respectively. The result indicated satisfactory internal consistency [17, 18].

**Fig 1:**
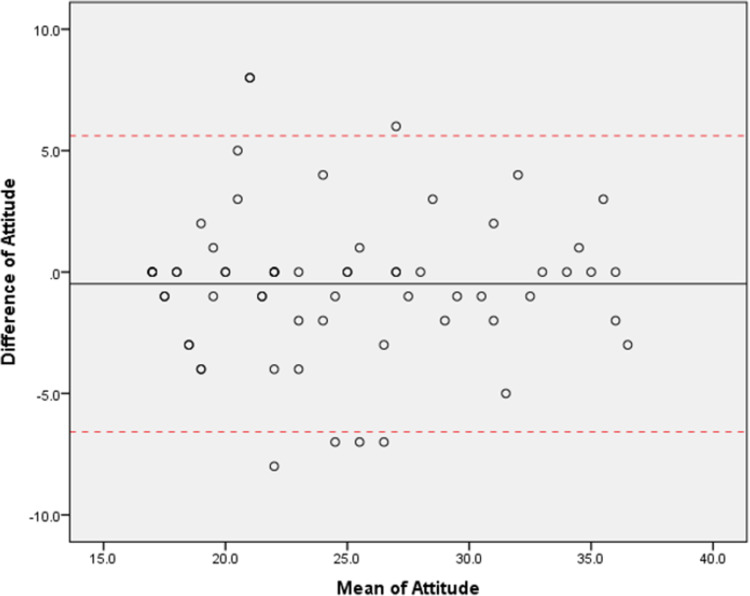
Blant & Altman plot for domain attitude.

**Fig 2:**
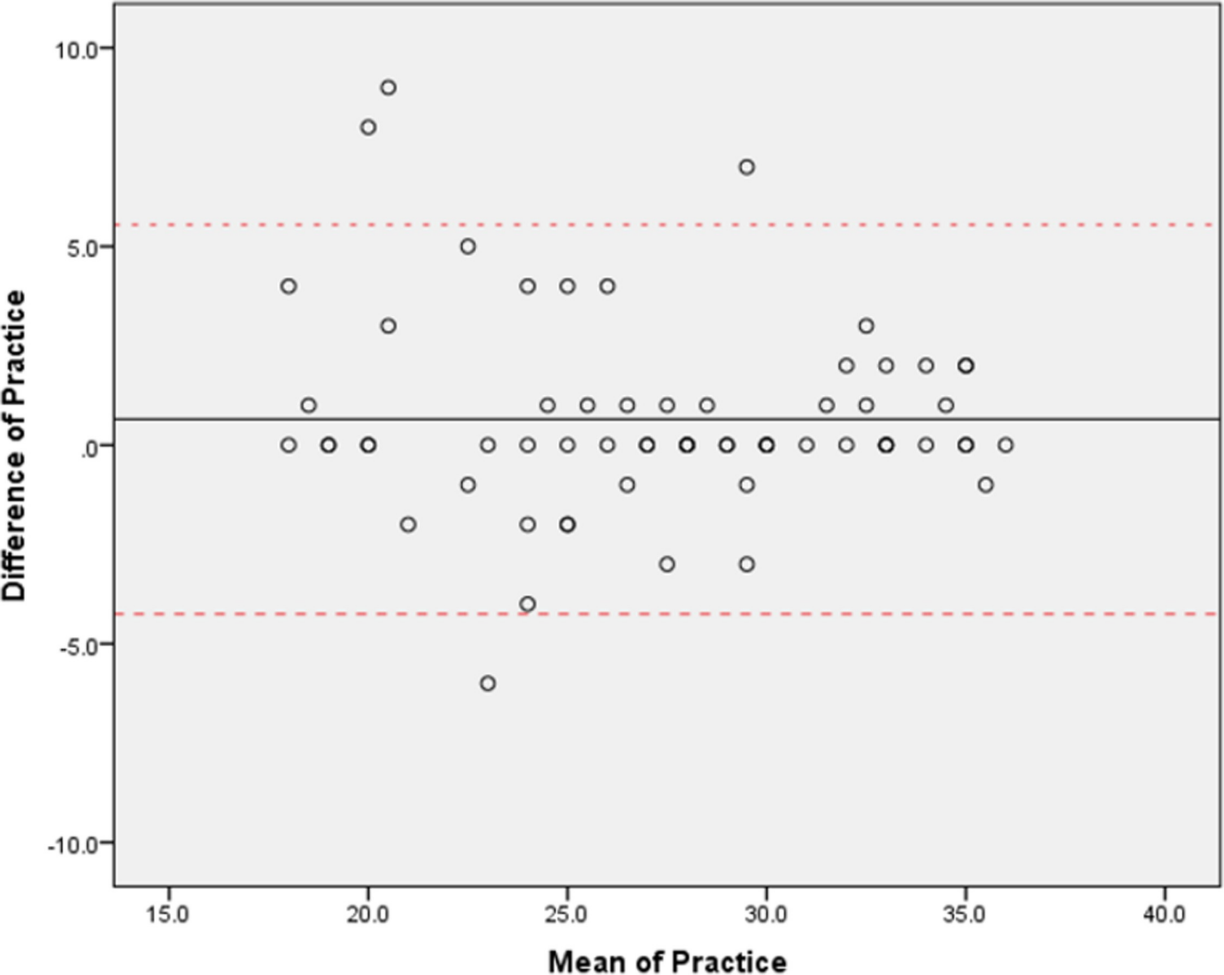
Blant & Altman plot for domain practice.

ICC results show a high degree of reliability for domain attitude and practice. The average measurement ICC was 0.926 with 95% CI [0.879, 0.955]; F (1,63) = 13.643, p<0.001 for domain attitude and the average measurement ICC was 0.938 with 95% CI [0.879, 0.936]; F (1,63) = 17.022, p<0.001 for domain practice. **Figs 1** and **2** shows the Bland and Altman plot of the 64 subjects in the study for domain attitude and practice, with 95% limits of agreement (LOA) and mean bias ±1.96SD for upper and lower LOA. The scatter plot graph shows no systematic bias for domain attitude and practice. The standard error of measurement (SEM) shows 0.46 and 0.28 for domain attitude and practice respectively. All the results show that the validation questionnaire is reliable over time.

## Discussion

The KAP questionnaire is widely accepted to investigate health-related behaviours and health-seeking practices. According to experts, the KAP questionnaire is appropriate for identifying baseline knowledge, misconception, attitude, belief, and behaviours in specific health topics [31]. To our knowledge, this is the first study that describes the construction of a validated questionnaire with psychometric properties for measuring the KAP of parents about vision problems among children.

In this study, we develop PEPC-KAPQ with 52 items under three domains: knowledge, attitude, and practice. The knowledge section consists of 26 items on a dichotomous scale emphasizing parents’ knowledge about vision problems among children. In contrast, the attitude and practice emphasize the parents’ behaviour towards vision problems among children. The attitude and practice consist of 17 and 9 items, respectively, with a 4-point Likert scale. The questionnaire takes about 15 to 20 minutes to complete. The PEP-KAPQ is the first measurement that fits the parents to assess parents’ knowledge, attitude and practice in eye problems among children.

The initial questionnaire was developed based on a systematic literature review followed by content validity to exclude those items that are problematic validity in terms of relevancy and clarity. Based on the content validity by five panels, two items in domain knowledge were eliminated because the items are problematic and did not represent the complete range of the attribute in domain knowledge and the objective of this study [17, 18]. The questions display excellent content validity for domain attitude and practice, and all the items remained. By performing face validity, the results show that the questionnaire is simple, clear, and easy to understand, indicating a good structure for the PEPC-KAPQ.

Construct validity and reliability were conducted to cover structure dimensions and determine internal consistency. According to explanatory factor analysis, three items of domain attitude and four items of domain practice were eliminated due to factor loading <0.30. The cumulative contribution of the other items on the corresponding factors that own enough factor loading (>0.30), means that the factors found in this analysis will be able to assess how well parents understand the concept of PEPC-KAPQ [27]. This result shows the close relationship between factors and items, indicate a good structure and acceptable psychometric properties of domain attitude and practice [17].

KR-20 and Cronbach’s *α* coefficient results reflect the internal consistency of the questionnaire. Our results show that the KR-20 for domain knowledge was 0.95, and Cronbach’s *α* for domain attitude and practice were 0.92 and 0.88, respectively, revealing that PEPC-KAPQ has good internal consistency reliability [18, 28]. This suggests that the items could well reflect the KAP of parents regarding common eye problems among children [16]. Bland and Altman plot result shows that the PEPC-KAPQ obtained an acceptable and satisfactory agreement for test-retest reliability. The results show that the validation questionnaire is reliable over time [32].

Our results show acceptable content, construct validities, and reliability of the questionnaire that indicate satisfactory properties and provide evidence for the psychometric properties of the questionnaire [18]. Therefore, the questionnaire is recommended to assess parents’ knowledge, attitude, and practice regarding eye problems among children. The findings from future studies can help develop strategies to increase the awareness of children’s eye problems among parents, hence reducing the risk of avoidable blindness among children.

The data collection was done in a single location, Kuala Lumpur, thus, the data collected and analyzed will only reflect urban population of the country. It is suggested that this study should be repeated in the rural population for the results to be a true representation of a national perspective. Another limitation of this study is the construct validity was assessed via the explanatory factor analysis thus, it is recommended to conduct a confirmatory factor analysis (CFA) to validate the KAP questionnaire.

## Conclusion

A new validated questionnaire for determining the KAP of parents about eye problems among children in the Malay language was developed. The final questionnaire consists of 52 items, with 26 items for domain knowledge, 17 for domain attitude and 9 for domain practice. Despite the limitations, this new instrument could lead to a better understanding of the current KAP that may help policymakers, health educators, clinicians, and researchers in identifying the barriers and misconceptions regarding child eye health care to reduce the prevalence of blindness among children by improving prevention strategies.

## Supporting information

S1 File (PDF)
