# Supplementary material for: Development and validation of parental knowledge, attitude and practice in eye problem among children questionnaire (PEPC-KAPQ)
Source: PLoS One. 2023 Sep 8;18(9):e0291062. doi: 10.1371/journal.pone.0291062 (PMC10490973; doi:10.1371/journal.pone.0291062)
Supplement: S1 File — (PDF) [file pone.0291062.s001.pdf]

UNIVERSITI KEBANGSAAN MALAYSIA
The National University of Malaysia

SOAL SELIDIK PENGETAHUAN, SIKAP DAN AMALAN IBU BAPA/PENJAGA MENGENAI MASALAH PENGLIHATAN DALAM KALANGAN KANAK-KANAK

1. BUTIRAN PERIBADI

Anda merupakan: □ Ibu □ Bapa □ Penjaga

Nama : ……………………………………..…………. Tarikh Lahir : …………...

Jantina : □ Lelaki □ Perempuan No Telefon : …………...

Bangsa □ Melayu □ Cina □ India □ Lain-lain (sila nyatakan) :………………………….

Tahap Pendidikan
□ Sekolah rendah
□ Sekolah menengah (PMR dan SPM)
□ STPM/ A-Level
□ Pendidikan tinggi (Kolej, Universiti)
□ Tiada pendidikan formal

Pendapatan Isi Rumah (bulanan) :………………………. Pekerjaan : ………………………...

Jumlah Anak :……………………….

Adakah anda/ahli keluarga mempunyai masalah penglihatan?

(Contoh : rabun jauh, rabun dekat, silau, mata juling, mata malas, selaput/katarak, dan lain-lain)

□ Ya, nyatakan :………………………..
□ Tidak
□ Tidak pasti

Soal selidik ini terdiri daripada **TIGA (3)** domain mengenai **Pengetahuan, Sikap dan Amalan** ibu bapa atau penjaga tentang masalah penglihatan dalam kalangan kanak-kanak. Jawapan yang anda beri menggambarkan ***Pengetahuan, Sikap dan Amalan*** tentang masalah penglihatan yang mungkin dialami oleh anak-anak anda.

1. *Sila jawab **SEMUA** soalan/pernyataan.*
2. *Tandakan √ pada setiap soalan/pernyataan.*

DOMAIN 1: PENGETAHUAN/ KNOWLEDGE

A: Tanda masalah penglihatan

**Adakah tanda-tanda berikut mempunyai kaitan dengan masalah penglihatan dalam kalangan kanak-kanak?**	**Ya**	**Tidak**
Anak mata berwarna putih		
Mata tidak selari/ Juling		
Mata hitam bergerak secara tidak terkawal (Nistagmus)		
Kerap menggosok mata		
Kerap mengadu sakit kepala		
Sentiasa mengerutkan dahi		
Sukar melihat jauh		
Membaca pada jarak yang dekat		
Tidak suka membaca/melakukan aktiviti dekat<br>(contoh: mewarna, melukis, bermain gajet dan sebagainya)		
Kepala teleng/senget apabila membaca/menonton televisyen		

B: Kesan masalah penglihatan

**Adakah tanda-tanda berikut merupakan kesan yang dialami oleh kanak-kanak apabila masalah penglihatan tidak dirawat?**	**Ya**	**Tidak**
Kesukaran belajar		
Tidak suka berkawan/menyendiri		
Buta		

C: Faktor penyebab masalah penglihatan

**Adakah faktor-faktor berikut boleh menyebabkan masalah penglihatan dalam kalangan kanak-kanak?**	**Ya**	**Tidak**
Kelahiran pra matang/ tidak cukup bulan		
Kurang aktiviti luar rumah		
Melakukan aktiviti dekat (contoh: membaca,melukis, penggunaan gajet dansebagainya) dalam jangka masa lama		
Kekurangan zat makanan		
Kecederaan/ kemalangan		

D: Kaitan masalah penglihatan dengan masalah kesihatan

**Adakah masalah berikut adalah penyakit mata/ masalah penglihatan dalam kalangan kanak-kanak?**	**Ya**	**Tidak**
Katarak (Selaput dalam mata)		
Rabun		
Ambliopia (Mata malas)		
Mata tidak selari/ Juling		

E: Rawatan atau pencegahan masalah penglihatan

	**Ya**	**Tidak**
Pemeriksaan mata sebelum memasuki alam persekolahan boleh mencegah masalah penglihatan yang lebih serius		
Masalah penglihtan dalam kalangan kanak-kanak boleh dirawat		
Doktor mata dan optometris sahaja layak untuk mengesahkan (diagnosis) masalah penglihatan kanak-kanak.		
Rawatan alternatif boleh merawat masalah penglihatan kanak-kanak		

DOMAIN 2: SIKAP/ ATTITUDE

	Sangat setuju	Setuju	Tidak setuju	Sangat tidak setuju
Anda berasa pemeriksaan mata adalah penting untuk anak anda.				
Anda berasa pemeriksaan mata perlu dilakukan secara berkala (sekurang-kurangnya setahun sekali) untuk anak anda.				
Anda berasa pemeriksaan mata boleh mencegah masalah penglihatan yang lebih serius kepada anak anda.				
Anda berasa bertanggungjawab membawa anak anda untuk pemeriksaan mata pada seawal usia sangat penting.				
Anda mengambil berat tentang masalah penglihatan anak anda.				
Anda akan membawa anak anda untuk pemeriksaan dan rawatan mata jika terdapat perubahan tingkah laku berkaitan masalah penglihatan.				
Anda akan memberikan anak anda memakai kaca mata seawal usia jika perlu.				
Anda akan membenarkan anak anda mengunakan ubat titis atau mendapatkan pembedahan seawal usia jika perlu.				
Anda mencari maklumat mengenai masalah penglihatan dalam kalangan kanak-kanak.				
Anda mendapatkan maklumat tentang masalah penglihatan melalui Internet (laman sesawang/media sosial/blog/*youtube*)				
Anda mendapatkan maklumat tentang masalah penglihatan melalui Majalah/surat khabar/buku ilmiah				
Anda mendapatkan maklumat tentang masalah penglihatan melalui Ceramah/pameran kesihatan				
Anda mendapatkan maklumat tentang masalah penglihatan melalui Keluarga/rakan-rakan				
Anda peka cara anak anda ketika melakukan aktiviti menggunakan jari/tangan.				
Anda peka cara anak anda ketika melakukan aktiviti menggunakan tangan dan kaki.				
Anda peka dengan cara anak anda memandang ketika berinteraksi dengan orang lain.				
Anda mengambil berat penguasaan kemahiran pembelajaran anak anda di sekolah.				

DOMAIN 3: AMALAN/ PRACTICE

**Berapa kerap anda…**	**Sangat kerap**	**Kerap**	**Jarang**	**Tidak pernah**
membawa anak anda untuk pemeriksaan mata?				
membawa anak anda mendapatkan rawatan mata alternatif (contoh: suplemen, rawatan tradisional, akupunktur dan sebagainya)?				
membawa anak anda untuk pemeriksaan mata walaupun tiada komplen/tanda?				
memberikan suplemen kepada anak anda untuk pencegahan masalah penglihatan?				
mengikut saranan doktor mata/optometris yang merawat masalah mata anak anda?				
mendapatkan maklumat berkenaan masalah penglihatan?				
membawa anak anda untuk mendapatkan kaunseling mengenai masalah penglihatan?				
mendapatkan khidmat nasihat/kaunseling mengenai masalah penglihatan anak anda?				
berasa tertekan dengan masalah penglihatan yang dihadapi oleh anak anda?				
